# Stereotactic versus hippocampal avoidance whole-brain radiotherapy combined with immune checkpoint inhibitors for multiple brain metastases in non-small cell lung cancer: a multi-center retrospective study

**DOI:** 10.3389/fonc.2026.1757781

**Published:** 2026-07-20

**Authors:** Xiaoliang Wang, Yanan Zheng, Aimin Wang, Xiaolan Zhao, Xudong Ruan, Choubin Wu, Chunquan Han

**Affiliations:** 1 Department of Radiotherapy, The Third Hospital of Zhangzhou, Zhangzhou, Fujian, China; 2 Department of Oncology, The Third Hospital of Zhangzhou, Zhangzhou, Fujian, China

**Keywords:** brain metastases, hippocampal avoidance whole-brain radiotherapy, immune checkpoint inhibitors, non-small cell lung cancer, stereotactic radiotherapy

## Abstract

**Objective:**

The optimal radiotherapy (RT) approach combined with immune checkpoint inhibitors (ICIs) for non-small cell lung cancer (NSCLC) patients with multiple brain metastases (BM) remains controversial. This study compared the clinical efficacy of stereotactic radiotherapy (SRT) plus ICIs versus hippocampal avoidance whole-brain radiotherapy (HA-WBRT) plus ICIs in this population.

**Methods:**

We retrospectively reviewed NSCLC patients with multiple BM(>4) who underwent RT plus ICIs from 2018 to 2024. Exclusion criteria included SRT-alone or WBRT-alone, Karnofsky performance status (KPS) <70, suspected leptomeningeal disease, or 1–4 BM lesions. Patients were divided into SRT+ICIs and HA-WBRT+ICIs cohorts by 1:1 propensity score matching (PSM). In the matched datasets, overall survival (OS) was the primary endpoint and intracranial progression-free survival (iPFS) was the secondary endpoint. Survival outcomes were analyzed using Kaplan-Meier and log-rank tests. Cox proportional hazards models identified factors associated with OS and iPFS.

**Results:**

Of the 256 patients diagnosed, 124 eligible patients (992 lesions) were analyzed. After 1:1 PSM, each cohort included 62 patients (SRT+ICIs and HA-WBRT+ICIs). The SRT+ICIs cohort demonstrated significantly longer median OS(21.4 vs. 15.5 months; P<0.001) and median iPFS (10.5 vs. 8.1 months; P<0.001) compared to the HA-WBRT+ICIs cohort. On multivariate analysis, SRT+ICIs was independently associated with improved OS (HR 0.381,95% CI 0.241-0.695, P<0.001) and iPFS (HR 0.513, 95% CI 0.297-0.961,P<0.001) versus HA-WBRT+ICIs. Adverse events (AEs) were similar between cohorts. No grade > 3 AEs were found.

**Conclusions:**

For NSCLC patients with multiple BM, SRT+ICIs is associated with significantly improved OS and iPFS versus HA-WBRT+ICIs, without an increased risk of AEs. SRT+ICIs represents a promising approach for this population.

## Introduction

Brain metastases (BM) are the most common malignant brain tumors, primarily originating from lung cancer, breast cancer, and melanoma (1, 2). About 20%-40% of lung cancer the patients develop BM, with 7%-10% presenting with BM at initial diagnosis (3, 4). Based on randomized trials demonstrating comparable overall survival (OS) alongside superior cognitive function and quality of life (QOL) outcomes, stereotactic radiosurgery or stereotactic radiotherapy (SRS/SRT) has become the optimal treatment for patients with 1–4 BM, supplanting whole-brain radiotherapy (WBRT) (5, 6). There is also growing evidence supporting SRS/SRT in selected patients with >4 brain metastases rather than implying that evidence is restricted to 1–4 lesions. Despite the lack of Level I evidence, SRT/SRS has been adopted as the first-line treatment for patients with relatively limited brain metastases in clinical practice (7, 8).

Although SRT/SRS is increasingly being used for multiple BM, WBRT retains a significant role. Especially, a more promising radiotherapy technique recognized by worldwide is Hippocampal Avoidance in WBRT planning. Hippocampal Avoidance whole brain radiotherapy(HA-WBRT) has emerged as a novel approach. This technique aims to minimize the radiation dose to the hippocampus, a region crucial for memory and cognitive function, while still delivering effective treatment to the rest of the brain. Several reports have demonstrated the feasibility and safety of HA-WBRT, showing promising results in preserving cognitive function without compromising tumor control (9–12). Therefore, HA-WBRT remains commonly utilized approach for multiple BM, further highlighting the ongoing controversy surrounding optimal treatment strategies for >4 metastases.

In recent years, immune checkpoint inhibitors (ICIs) have shown promise in treating BM, particularly when combined with local therapies. Both SRS/SRT and ICIs individually improve OS in patients with (oligo)metastatic solid tumors. Furthermore, the combination of local therapies and ICIs has demonstrated encouraging outcomes in several reports (13–18). While combining ICIs with SRS/SRT holds promise for enhanced efficacy in BM management, concerns exist regarding a potentially increased risk of neurotoxicity and impact on QOL (19–21). Although ICIs alone show good response rates in melanoma patients with BM, response rates for BM treated solely with ICIs are generally only around 30%, significantly lower than the 90-95% response rates observed when combined with SRS/SRT (22, 23). Meanwhile, The beneficial combination effect of ICIs and SRS/SRT observed in metastatic melanoma has also been confirmed in lung cancer patients with BM. It is hypothesized that the high-precision, high-dose characteristics of SRT may induce immunogenic cell death more effectively, potentially leading to a stronger synergistic effect with ICIs. Currently, direct comparisons of the clinical efficacy between SRT+ICIs and HA-WBRT+ICIs are scarce. In this retrospective cohort study, we compared clinical outcomes in patients with more than four brain metastases (BM) who received concurrent SRT+ICIs versus those treated with HA-WBRT+ICIs, with 1:1 propensity score matching (PSM) applied to balance baseline characteristics between the two groups.

## Methods

### Patient selection

We retrospectively reviewed NSCLC patients with multiple BM who underwent SRT or WBRT between 2018 and 2024 from three institutional disease databases (The Third Hospital of Zhangzhou, XiaMen ChangGung Hospital, Army 73rd Group Military Hospital). Approval was obtained from the institutional review board of each participating institution, and all patients signed the informed consent forms and withdrawal forms that declared participants were included unless they explicitly decide to voluntarily withdraw. All analyses were implemented in accordance with the relevant guidelines and regulations.

Inclusion criteria were: (1) Age ≥18 years; (2) Pathologically confirmed NSCLC; (3) multiple BM (>4 lesions) confirmed by magnetic resonance imaging(MRI); (4) ICIs treatment initiated ≤ 2 weeks before or after RT; (5) KPS score ≥70; (6) Treated with RT+ICIs. Exclusion criteria were: underwent WBRT+ICIs without hippocampal avoidance, Karnofsky Performance Status(KPS) score (KPS)≤60, treatment with SRT alone, prior neurosurgery, cranial radiation history, concurrent targeted therapy, suspected leptomeningeal disease (LMD), and receipt of ICIs > 2 weeks before or after RT initiation.

### Therapeutic regimen

All eligible patients were propensity-score matched (1:1) into two cohorts: SRT+ICIs and HA-WBRT+ICIs. Imaging and Target Delineation: For SRT planning, thin-slice contrast-enhanced MRI (post-contrast T1-weighted sequences) was mandatory. All visible metastatic lesions were contoured as gross tumor volumes (GTVs) on fused axial, sagittal, and coronal images with a slice thickness of 1.5 mm. Target volume delineation for every patient was reviewed in a multidisciplinary setting consisting of radiation oncologists, diagnostic radiologists, and neurosurgeons. SRT+ICIs Cohort: The planning target volume (PTV) was defined as the GTV plus a 2-mm isotropic margin. SRT was delivered at 30–36 Gy in 5–6 fractions (6 Gy per fraction). HA-WBRT+ICIs Cohort: The clinical target volume (CTV) included the whole brain parenchyma excluding the hippocampal avoidance region (HAR), which was defined as the hippocampal structure expanded by a 7-mm three-dimensional margin with dose limits of Dmax ≤ 14 Gy and Dmean ≤ 9 Gy. The planning clinical target volume (PCTV) was defined as the CTV plus a 2-mm isotropic margin. HA-WBRT delivered 30 Gy in 10–12 fractions (2.5-3.0 Gy per fraction, 5 fractions per week). Treatment Planning: All plans were generated using volumetric modulated arc therapy (RapidArc, Varian Medical Systems). ICIs Therapy: All patients received ≥ 2 cycles of ICIs treatment within ≤2 weeks before or after the initiation of SRT. No patients were administered memantine.

### Follow-up

OS was defined as the period from the initiation of SRT+ICIs or HA-WBRT+ICIs treatment until death from any cause or the last follow-up. Intracranial progression-free survival (iPFS) was defined as the duration from the treatment initiation until death or documented intracranial progression (Intracranial progressive disease was confirmed on follow-up contrast-enhanced cranial MRI in accordance with the RECIST 1.1 criteria). The following baseline data were collected: age, gender, KPS, histological type, number of BM, maximum diameter of the largest BM, presence of Central nervous system(CNS) symptoms, extracranial metastases (ECM), and graded prognostic assessment score (GPA) at BM diagnosis. All patients underwent testing for epidermal growth factor receptor (EGFR) mutations, anaplastic lymphoma kinase (ALK) rearrangement, and programmed death-ligand 1 expression. All eligible patients were assigned into two cohorts: the SRT+ICIs and the HA-WBRT+ICIs cohorts, and potential prognostic factors between cohorts were balanced using 1:1 PSM. In the matched cohort, the primary endpoint was OS, with iPFS as the secondary endpoint. The patient follow-up concluded on December 31, 2024.

### Statistical analysis

Statistical analysis were performed using SPSS 26.0 software. Baseline characteristics between cohorts were compared using the chi-square test (categorical variables) and analysis of variance (continuous variables). OS and iPFS were estimated via Kaplan-Meier analysis, with between-group comparisons assessed by the log-rank test. Univariate and multivariate Cox proportional hazards regression analyses were performed to identify patient and treatment variables associated with OS and iPFS within each cohort. Statistical significance was defined as a two-sided p -value < 0.05.

## Results

### Patient baseline characteristics

**Figure 1:**
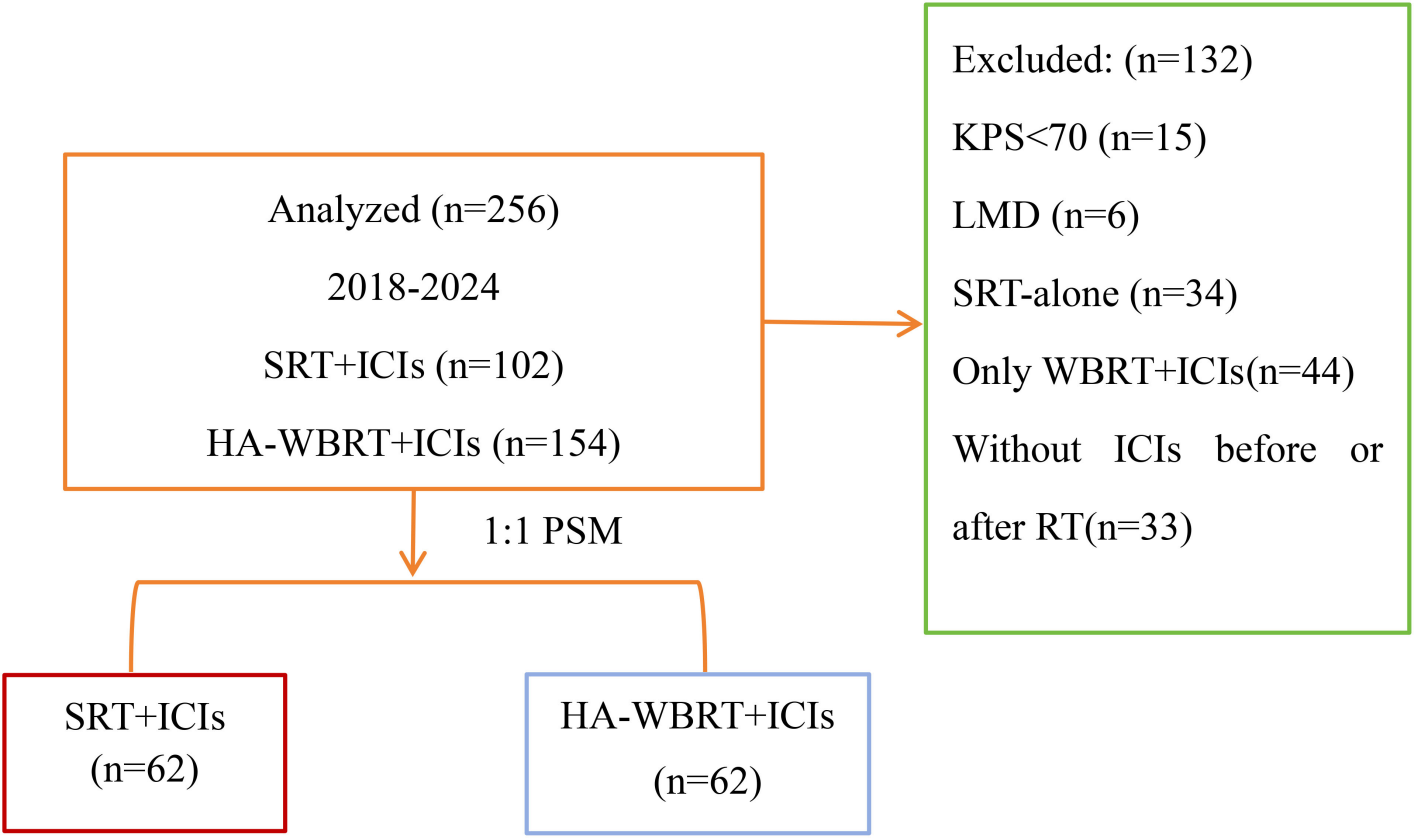
The consort diagram of this study. BM brain metastases, KPS Karnofsky performance status, LMD leptomeningeal disease, HA hippocampal avoidance, WBRT whole-brain radiotherapy, PSM propensity score matching, SRT stereotactic radiotherapy, ICI immune Checkpoint Inhibitor.

Of the 256 patients diagnosed, 124 eligible patients with 992 lesions were analyzed, and 132 patients were ineligible. 62 patients were in the SRT+ICIs group and HA-WBRT+ICIs group, respectively (**Figure 1**). A total of 132 patients were excluded (underwent WBRT+ICIs without hippocampal avoidance in 44, KPS ≤ 60 in 15, treatment with SRT alone in 34, LMD in 6, and without ICIs before or after RT 33). The characteristics of all patients in this study are shown in **Table 1**.The SRT+ICIs group tended to have some biased features in the original data, for example a worse GPA score(SMDs=0.413), more NSCLC adenocarcinoma(Ad, SMDs=0.435) with EGFR/ALK (SMDs=0.476) or PD-L1positive (SMDs=0.151), younger patients (SMDs=0.303), extra-cranial disease control at RT initiation (SMDs=0.136), and No. of BM (SMDs=0.512).

**Table 1:** Patient characteristics.

Characteristics	Raw dataset (n=256)			Matched dataset (n=124)		
	SRT+ICIs(%)	WBRT+ ICIs (%)	SMDs	SRT+ICIs(%)	HA-WBRT+ICIs(%)	SMDs
Sample size, No	102	154		62	62	
Gender						
Male	63 (61.8)	96 (62.3)	0.066	39 (62.9)	40 (64.5)	0.052
Female	39 (38.2)	58 (37.7)		23 (37.1)	22 (35.5)	
Age, year	62 (31-86)	60 (28-85)	0.303	61 (31-83)	62 (30-81)	0.056
<70				45 (72.6)	42 (67.7)	0.050
≥70				17 (27.4)	19 (32.3)	
KPS						
100	9 (8.8)	7 (4.5)	0.091	5 (8.1)	4 (6.5)	0.087
90	46 (45.1)	76 (49.4)		33 (53.2)	31(50.0)	
80	30(29.4)	43 (27.9)		21 (33.9)	24 37.0)	
70	11 (10.8)	19 (13.0)		3 (4.8)	4 (6.5)	
60	6 (5.9)	9 (5.2)				
No. of BM at BM diagnosis						
5-10	70 (68.6)	71 (46.1)	0.512	43 (69.4)	41 (66.1)	0.081
11-22	32 (31.4)	83(53.9)		19 (30.6)	21 (33.9)	
Diameter of the largest tumor, mm						
<10	25 (24.5)	36 (23.4)	0.093	19 (30.6)	17 (27.4)	0.086
10-19	26 (25.5)	40 (26.0)		15 (24.2)	14 (22.6)	
20-29	39 (38.2)	59 (38.3)		23 (37.1)	25 (40.3)	
≥30	12 (11.8)	19 (12.3)		5 (8.1)	6 (9.7)	
GPA						
0-1.0	22 (22.7)	49 (31.8)	0.413	14 (22.6)	16 (25.8)	0.082
1.5-2.0	37 (37.3)	70 (45.5)		26 (41.9)	25 (40.3)	
2.5-3.0	43 (40.0)	35 (22.7)		22 (35.5)	21 (33.9)	
3.5-4.0	0	0		0	0	
Neurologic symptoms Positive	19 (18.6)	31 (20.1)	0.043	13 (21.0)	15 (24.2)	0.025
Histology (%)						
SCC	38 (37.2)	65 (42.2)	0.435	29 (46.8)	31 (50.0)	0.079
Ad	62 (60.8)	69 (44.8)		33 (53.2)	31 (50.0)	
other^†^	2 (2.0)	20 (13.0)		0	0	
Extra-Cranial Disease Control at time of RT						
controlled	62 (60.8)	72 (46.8)	0.136	41 (66.1)	42 (67.7)	0.049
uncontrolled	40 (39.2)	82 (53.2)		21 (33.9)	20 (32.3)	
PD-L1 status						
50-100%	17 (16.0)	21 (13.6)	0.151	13 (21.0)	14 (22.6)	0.042
1-49%	38(37.3)	34 (22.1)		18 (29.0)	19 (30.6)	
Negative	47 (46.7)	99 (64.3)		31 (50.0)	29 (46.8)	
Mutations						
EGFR/ALK	47 (46.1)	35 (22.7)	0.476	27 (43.5)	28 (45.2)	0.068
Negative/other	55 (53.9)	119 (77.3)		35 (56.5)	34 (54.8)	
Chemotherapy						
Yes	23 (22.5)	53 (34.4)	0.108	8 (12.9)	7 (9.7)	0.070
No	79 (77.5)	101 (65.6)		54 (87.1)	55 (90.3)	
The timing of ICIs						
Before SRT	42 (41.2)	76 (49.4)	0.066	19 (30.6)	17 (27.4)	0.032
After SRT	60 (58.2)	78 (50.6)		43 (69.4)	45 (62.6)	
Immunotherapy						
Pembrolizumab	32 (31.4)	26 (16.9)	0.118	32 (51.6)	26 (41.9)	0.043
Atezolizumab	23 (22.5)	29 (18.8)		22 (35.5)	29 (46.8)	
Trelilizumab	11 (10.8)	22 (14.3)		3 (4.8)	4 (6.5)	
Sintilimab	12 (11.8)	21 (13.6)		5 (8.1)	3(4.8)	

BM, brain metastases; GPA, graded prognostic assessment; KPS, Karnofsky Performance Status; ECM, extracranial metastases; EGFR, epidermal growth factor receptor; ALK, anaplastic lymphoma kinase; PD-L1, programmed death-ligand 1; SRT, stereotactic radiotherapy; ICI, immune Checkpoint Inhibitor; RT, radiation therapy SCC, squamous cell carcinoma; Ad, adenocarcinoma; No., Number; HA-WBRT, hippocampal avoidance whole-brain radiation therapy.

**Figure 2:**
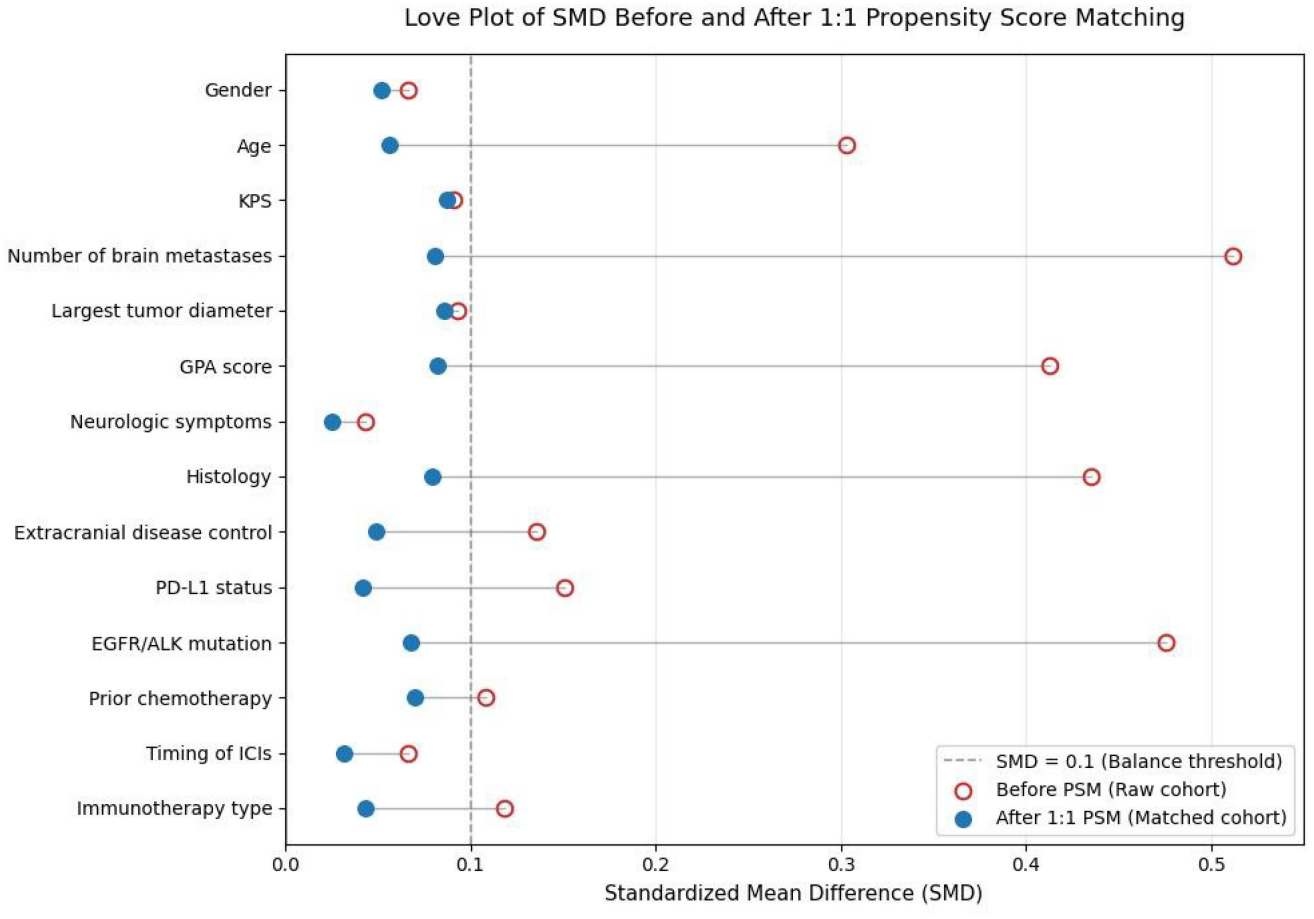
Love plot displaying standardized mean differences (SMDs) of all baseline covariates before and after 1:1 propensity score matching (PSM). Hollow red circles represent SMD values in the raw overall cohort, and solid blue circles indicate SMD values in the matched cohort. Horizontal gray lines connect the pre- and post-matching SMD of each variable. The vertical dashed gray line at SMD = 0.1 denotes the threshold for acceptable baseline balance. All covariates achieved SMD < 0.1 after matching, indicating satisfactory balance between the SRT+ICIs and HA-WBRT+ICIs groups.

All patients were divided into two cohorts (SRT+ICIs and HA-WBRT+ICIs groups) through 1:1 propensity score matched (**Figure 2**). **Table 1** showed that all characters were well balanced in the matched cohorts, and the average number of lesions per patient in the matched cohort was 8. The median lesions was 6 in the SRT+ICIs group and 7 in the HA-WBRT+ICIs group. All patients had not received local treatment for brain metastasis before enrollment. The proportions of 11–22 lesions were 30.6% in the SRT+ICIs group and 33.9% in the HA-WBRT+ICIs group. No cases with a GPA of 3.5-4.0 were shown in the entire dataset. In the matched dataset, 21.0% of patients in the SRT+ICIs group and 24.2% in the HA-WBRT+ICIs group exhibited positive neurological symptoms. Of the patients with Ad, 53.2% were in the SRT+ICIs group and 50.0% in the HA-WBRT+ICIs group. EGFR/ALK mutations were detected in 43.5% and 45.2% of patients in the SRT+ICIs and HA-WBRT+ICIs cohorts, respectively. Likewise, positive PD-L1 expression was identified in 50.0% of patients in the SRT+ICIs cohort and 53.2% of those in the HA-WBRT+ICIs cohort. ICIs combined with chemotherapy were 8 in the SRT+ICIs group and 7 in the HA-WBRT+ICIs group.19 patients administrated ICIs before SRT in the SRT+ICIs group and 17 patients in the HA-WBRT+ICIs group who were given ICIs prior to HA-WBRT. The other patients received ICIs after SRT.

### Overall survival and iPFS outcomes analysis

**Figure 3:**
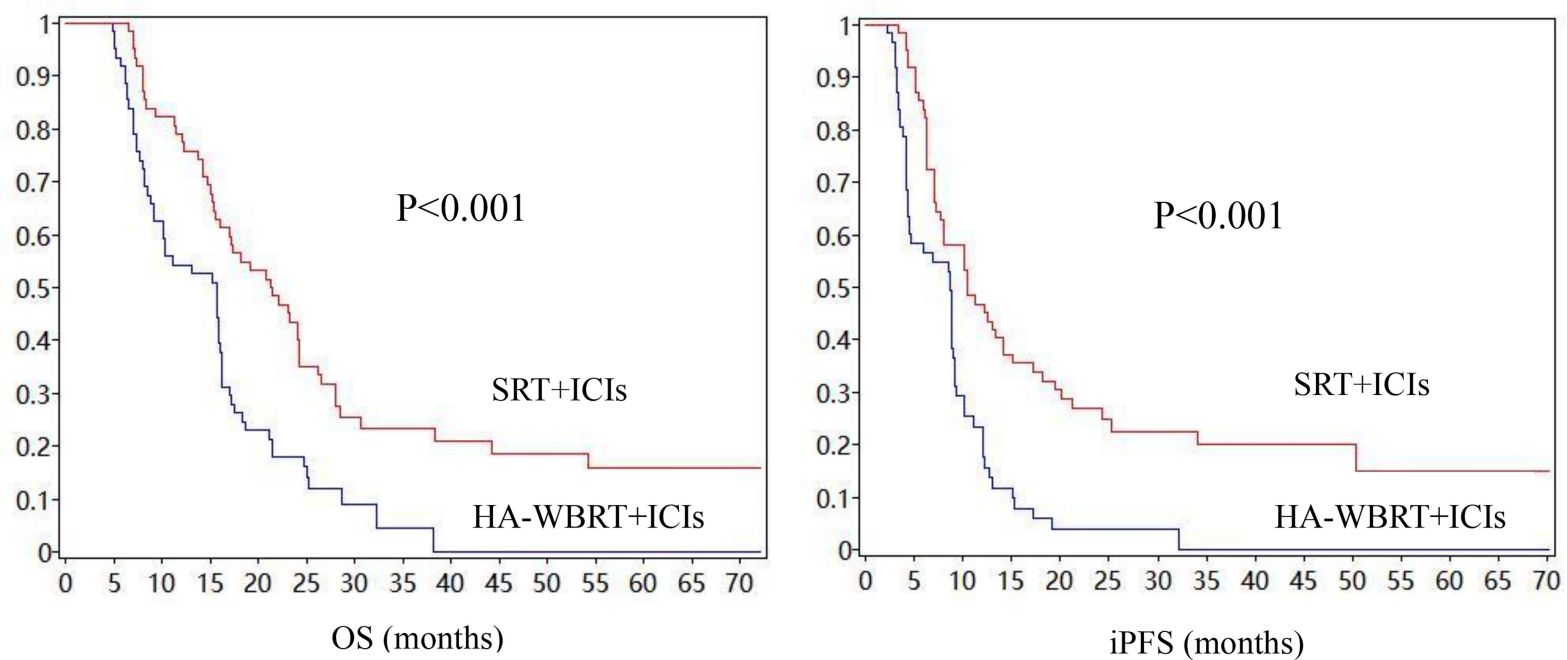
Kaplan-Meier survival curves for overall survival (OS) and intracranial progression-free survival (iPFS) comparing the SRT+ICIs group to the hippocampal avoidance whole-brain radiotherapy (HA-WBRT+ICIs) group in the propensity score-matched dataset. SRT stereotactic radiotherapy, ICI immune Checkpoint Inhibitor.

At the final follow-up in the matched cohort, 36 deaths and 49 intracranial progression occurred in the SRT+ICIs group, compared to 48 deaths and 51 intracranial progression in the HA-WBRT+ICIs group. The median follow-up time was 31 months in the matched datasets. Patients receiving SRT+ICIs demonstrated significantly longer median OS (21.4 months, 95% CI 20.1–28.1) than those receiving HA-WBRT+ICIs (15.5 months, 95% CI 12.4–16.3; P < 0.001; **Figure 3**). Multivariate analysis was performed after adjustment for imbalanced baseline covariates, including KPS>80, age <70 years, presence of CNS symptoms, Ad, controlled extracranial metastases at RT initiation, PD-L1 ≥ 50%, EGFR/ALK positivity, and the treatment regimen of SRT+ICIs were significantly associated with improved OS (**Table 2**).

**Table 2:** Univariate and multivariate analyses of variables associated with overall survival.

All patients of 1:1 PSM n=124						
Variable	Univariate analysis			Multivariate analysis		
	HR	95% CI	P	HR	95% CI	P
Age(<70 vs ≥70)	0.672	0.541-0.932	0.014	0.771	0.438-0.965	0.022
Gender(Male vs Female)	1.143	0.826-1.935	0.083			
KPS						
70/80 vs 90/100	22.214	5.221-83.312	0.002	11.157	1.911-66.342	0.006
No. of BM at BM diagnosis						
5–10 vs 11-22	1.321	0.742-1.598	0.632			
Diameter of the largest tumor, mm						
<10 vs 10-19	1.124	0.782-1.893	0.164			
<10 vs 20-29	1.253	0.801-1.839	0.241			
<10 vs ≥30	1.113	0.496-2.082	0.911			
GPA						
0-1.0 vs 1.5-2.0	0.394	0.237-0.688	0.003			
0-1.0 vs 2.5-3.0	0.335	0.176-0.521	<0.001			
Neurologic symptoms						
Positive vs Negative	2.021	1.114-3.241	0.006	1.021	1.025-2.723	0.011
Histology (%)						
SCC VS Ad	1.142	0.321-1.741	0.671	3.411	1.412-9.611	0.012
ECM Control at time of RT						
Controlled vs uncontrolled	2.123	1.122-5.411	0.041	2.814	1.231-5.832	0.025
PD-L1 status						
50-100% vs Negative	0.398	0.213-0.562	<0.001	0.042	0.208-0.718	0.011
1-49% vs Negative	0.891	0.522-1.521	0.84			
Mutations						
EGFR/ALK vs Negative/other	0.432	0.298-0.623	0.005	0.443	0.284-0.656	0.012
Regimens						
SRT+ICIs vs HA-WBRT+ICIs	0.312	0.129-0.478	0.016	0.381	0.214-0.695	<0.001

BM, brain metastases; GPA, graded prognostic assessment; KPS, Karnofsky Performance Status; ECM, extracranial metastases; EGFR, epidermal growth factor receptor; ALK, anaplastic lymphoma kinase; PD-L1, programmed death-ligand 1; SRT, stereotactic radiotherapy; ICI, Immune Checkpoint Inhibitor; SCC, squamous cell carcinoma; Ad, adenocarcinoma; No., Number; HA-WBRT+ICI, hippocampal avoidance whole-brain radiation therapy.

In the original datasets, while a trend toward longer iPFS was observed for SRT+ICIs (P = 0.045), a statistically significant difference in median iPFS was confirmed in the matched cohort (10.5 months for SRT+ICIs vs. 8.1 months for HA-WBRT+ICIs; P < 0.001; **Figure 3**). Multivariate analysis was performed after adjustment for imbalanced baseline covariates, including KPS > 80, presence of CNS symptoms, controlled extracranial metastases at RT initiation, PD-L1 ≥ 50%, and the treatment regimens of SRT+ICIs were significantly correlated with improving iPFS (**Table 3**). In the matched datasets, the sites of first intracranial progression among the 49 events in the SRT+ICIs group were: local failure (n=4, 8.2%), distant intracranial failure (n=39, 79.6%), and both local and distant failure (n=6, 12.2%). Among the 51 events in the HA-WBRT+ICIs group, failure sites were: local only (n=10, 19.6%), distant only (n=36, 70.6%), and both (n=5, 9.8%) (**Supplementary Table 1**), 12 patients with intracranial failure received salvage SRT.

**Table 3:** Univariate and multivariate analyses of variables associated with intracranial progression-free survival.

All patients of 1:1 PSM n=124						
Variable	Univariate analysis			Multivariate analysis		
	HR	95% CI	P	HR	95%	P
Age(<70 vs ≥70)	0.913	0.589-1.437	0.553			
Sex (Male vs Female)	1.314	0.779-1.613	0.441			
KPS						
70/80 vs 90/100	18.223	4.632-44.176	0.013	10.762	2.443-36.811	0.036
No. of BM at BM diagnosis						
5–10 vs 11-22	1.652	0.776-1.436	0.813			
Diameter of the largest tumor, mm						
<10 vs 10-19	1.233	0.817-1.743	0.092			
<10 vs 20-29	1.116	0.714-1.522	0.895			
<10 vs ≥30	1.356	0.731-1.893	0.612			
GPA						
0-1.0 vs 1.5-2.0	0.589	0.453-0.872	<0.001			
0-1.0 vs 2.5-3.0	0.557	0.415-0.911	0.006			
Neurologic symptoms						
Positive vs Negative	2.152	1.321-3.642	0.004	2.005	1.125-3.143	0.012
Histology (%)						
SCC VS Ad	1.213	0.479-1.968	0.845			
ECM Control at time of RT						
Controlled vs uncontrolled	2.554	1.332-5.668	0.035	3.651	1.655-6.443	0.028
PD-L1 status						
50-100% vs Negative	0.457	0.332-0.779	<0.001	0.532	0.311-0.674	0.003
1-49% vs Negative	0.774	0.502-1.224	0.077			
Mutations						
EGFR/ALK vs Negative/other	0.611	0.432-0.902	0.011	0.562	0.354-0.876	0.043
Regimens						
SRT+ICIs vs HA-WBRT+ICIs	0.451	0.264-0.910	0.025	0.513	0.297-0.961	<0.001

BM, brain metastases; GPA, graded prognostic assessment; KPS, Karnofsky Performance Status; ECM, extracranial metastases; EGFR, epidermal growth factor receptor; ALK, anaplastic lymphoma kinase; PD-L1, programmed death-ligand 1; SRT, stereotactic radiotherapy; ICI, Immune Checkpoint Inhibitor; SCC, squamous cell carcinoma; Ad, adenocarcinoma; No., Number; HA-WBRT, hippocampal avoidance whole-brain radiation therapy.

### Treatment-related adverse events

Adverse events (AEs) were evaluated in accordance with the Common Terminology Criteria for Adverse Events Version 5.0 (CTCAE v5.0). Treatment-related adverse events (TRAEs) were documented in 16 patients (12.9%), including 9 patients with Grade 1 AEs (7.3%), 6 patients with Grade 2 AEs (4.8%), and 1 patient with Grade 3 AEs (0.8%). No statistically significant intergroup difference was detected in the incidence of all-grade AEs (P > 0.05), and no Grade ≥4 adverse events were reported during follow-up.

One patient (0.8%) in the HA-WBRT+ICIs cohort developed intracranial tumor hemorrhage at 3 weeks post-treatment. Grade 1–2 radiation necrosis occurred in 5 patients (4.0%) at a median interval ranging from 6.5 to 34 months after radiotherapy: 3 patients in the SRT+ICIs cohort (2 with Grade 1, 1 with Grade 2) and 2 patients in the HA-WBRT+ICIs cohort (1 with Grade 1, 1 with Grade 2). All five patients were treated with steroids for 4 to 8 weeks, and lesions resolved completely without additional invasive interventions.

Grade 1–2 neurocognitive dysfunction was observed in 6 patients (4.8%) at 8.7 to 38 months after treatment, consisting of 2 patients from the SRT+ICIs cohort (both Grade 1) and 4 patients from the HA-WBRT+ICIs cohort (3 Grade 1, 1 Grade 2). Grade 1–2 seizures developed in 4 patients, with 2 patients in the SRT+ICIs cohort (1 Grade 1, 1 Grade 2) and 2 patients in the HA-WBRT+ICIs cohort (both Grade 2). No significant difference in hematological toxicity was found between the two cohorts (P = 0.562). Detailed hematological toxicity data were not collected in this retrospective study.

## Discussion

This study aimed to compare the clinical efficacy of concurrent SRT+ICIs with HA-WBRT+ICIs in the treatment of NSCLC patients with multiple BM. After rigorous adjustment for key prognostic factors via 1:1 PSM, the SRT+ICIs regimen demonstrated significantly improved median OS (21.4 vs. 15.5 months) and iPFS (10.5 vs. 8.1 months) compared to HA-WBRT+ICIs. These results provide valuable insights, offering one of the first comparative evaluations of SRT+ICIs specifically for multiple BM. Crucially, the analysis included detailed patient characteristics, encompassing systemic treatment history and targetable gene mutations, which are critical confounders influencing survival outcomes. The data indicated that the incidence of treatment-related adverse events was similar in both groups, and there was no significant difference in the intracranial recurrence rate (P = 0.318, **Supplementary Table 1**).

In recent years, immune checkpoint inhibitors (ICIs) have shown promise in treating brain metastases, particularly when combined with local therapies. RT and ICIs, used separately, both have indicated an improved overall survival in BM. The rationale for combining RT and ICIs extends beyond RT’s direct cytotoxic effects. SRT induces immunogenic cell death and modifies the tumor microenvironment, potentially enhancing systemic anti-tumor immunity and contributing to the rare but significant abscopal effect, particularly relevant with highly focused SRT/SRS. To date, SRT/SRS+ICIs has gradually been used for patients with multiple brain metastases in clinical practice, despite the lack of level I evidence (8). Furthermore, several reports showed the encouraging outcomes of the combination of SRT/SRS and ICIs for BM (4–11).

The superior OS observed in patients receiving SRT plus ICIs may be attributable to multiple mechanisms. First, SRT delivers a high biologically effective dose, which robustly triggers immunogenic cell death. When combined with ICIs, this effect can further potentiate systemic antitumor immune responses. Second, SRT achieves exceptional sparing of normal brain tissue, particularly the hippocampal region, thereby minimizing damage to neural stem cells-a known detriment of WBRT. Such favorable neuroprotective properties help preserve cognitive function, improve long-term quality of life, and may ultimately confer a survival advantage. However, the focal nature of SRT inherently limits its effectiveness against microscopic metastatic disease, likely explaining the slightly higher rate of intracranial distant failure (IDF) observed in the SRT+ICIs cohort. Of note, this between-group difference did not reach statistical significance in our study. (P = 0.318, **Supplementary Table 1**).

Conversely, while the HA-WBRT+ICIs regimen yielded inferior OS and iPFS outcomes, it demonstrated superior control over microscopic disease, reflected in its slightly lower IDF rate (24). Regarding safety, a notable finding was the slightly higher incidence of symptomatic radiation necrosis (RN) in the SRT+ICIs group. This suggests that the cumulative radiation dose delivered to multiple lesions with SRT, even with advanced planning techniques, remains a risk factor for RN. Importantly, ICIs may potentiate this risk by enhancing localized immune-inflammatory responses within irradiated tissue.

Several limitations warrant consideration. Primarily, the retrospective design, despite employing rigorous propensity score matching to minimize confounding, remains susceptible to residual selection bias.

Our study supports a more individualized treatment strategy for NSCLC patients with multiple BM. In patients with relatively limited brain metastases (maximum quantity not found), longer anticipated survival, and high emphasis on quality of life, aggressive SRT+ICIs may be the preferred option, though it requires acceptance of a slightly higher risk of radiation necrosis and close follow-up. Conversely, for patients with diffuse, numerous BM and rapid disease progression, HA-WBRT+ICIs provides more reliable whole-brain control, representing a balanced choice between efficacy and quality of life. Future prospective randomized trials are warranted to definitively establish the roles of these two strategies.

## Conclusions

Within the therapeutic context of ICIs for NSCLC patients with multiple BM, both SRT+ICIs and HA-WBRT+ICIs represent effective combinatorial strategies. Concurrent SRT and ICIs are associated with significantly improved OS and iPFS versus HA-WBRT+ICIs, without increase risk of AEs. SRT combined with ICIs represents a promising approach for this population.

## Data Availability

The original contributions presented in the study are included in the article/**Supplementary Material**. Further inquiries can be directed to the corresponding author.

## References

[1] Mitchell DK Kwon HJ Kubica PA Huff WX O'Regan R Dey M . Brain metastases: An update on the multi-disciplinary approach of clinical management. Neurochirurgie. (2022) 68:69–85. doi: 10.1016/j.neuchi.2021.04.001 33864773 PMC8514593

[2] Giantini-Larsen AM Juthani RG Pannullo SC Knisely JPS . Novel approaches to the management of patients with 5-15 brain metastases: a narrative review. Chin Clin Oncol. (2022) 11:17. doi: 10.21037/cco-22-15 35534795

[3] Page S Milner-Watts C Perna M Janzic U Vidal N Kaudeer N . Systemic treatment of brain metastases in non-small cell lung cancer. Eur J Cancer. (2020) 132:187–98. doi: 10.1016/j.ejca.2020.03.006 32380429

[4] Pellerino A Bruno F Rudà R Soffietti R . Systemic therapy for lung cancer brain metastases. Curr Treat Options Oncol. (2021) 22:110. doi: 10.1007/s11864-021-00911-7 34693454

[5] Nabors LB Portnow J Ahluwalia M Baehring J Brem H Brem S . Central nervous system cancers, version 3.2020, NCCN clinical practice guidelines in oncology. J Natl Compr Canc Netw. (2020) 18:1537–70. doi: 10.6004/jnccn.2020.0052 33152694

[6] Horbinski C Nabors LB Portnow J Baehring J Bhatia A Bloch O . NCCN Guidelines® Insights: Central nervous system cancers, version 2.2022. J Natl Compr Canc Netw. (2023) 21:12–20. doi: 10.6004/jnccn.2023.0002 36634606

[7] Aizer AA Shin KY Catalano PJ Ricca I Johnson M Benham G . Treatment for brain metastases with stereotactic radiation vs hippocampal-avoidance whole brain radiation: a randomized clinical trial. JAMA. (2026) 335(7):663–673. doi: 10.1001/jama.2026.0076 41712219 PMC12921566

[8] Yamamoto M Serizawa T Shuto T Akabane A Higuchi Y . Stereotactic radiosurgery for patients with multiple brain metastases (JLGK0901): a multi-institutional prospective observational study. Lancet Oncol. (2014) 15:387–95. doi: 10.1016/S1470-2045(14)70061-0 24621620

[9] Gondi V Hermann BP Mehta MP . Hippocampal dosimetry predicts neurocognitive function impairment after fractionated stereotactic radiotherapy for benign or low-grade adult brain tumors. Int J Radiat Oncol Biol Phys. (2012) 83:e487–93. doi: 10.1016/j.ijrobp.2011.10.021 22209148 PMC3462659

[10] Gondi V Pugh SL Tome WA Caine C Corn B Kanner A . Preservation of memory with conformal avoidance of the hippocampal neural stem-cell compartment during whole-brain radiotherapy for brain metastases (RTOG 0933): a phase II multi-institutional trial. J Clin Oncol. (2014) 32:3810–6. doi: 10.1200/JCO.2014.57.2909 25349290 PMC4239303

[11] Brown PD Gondi V Pugh S Tome WA Wefel JS Armstrong TS . Hippocampal avoidance during whole-brain radiotherapy plus memantine for patients with brain metastases: Phase III trial NRG Oncology CC001. J Clin Oncol. (2020) 38:1019–29. doi: 10.1200/JCO.19.02767 32058845 PMC7106984

[12] Gondi V Deshmukh S Brown PD Wefel JS Armstrong TS Tome WA . Sustained preservation of cognition and prevention of patient-reported symptoms with hippocampal avoidance during whole-brain radiation therapy for brain metastases: Final results of NRG Oncology CC001. Int J Radiat Oncol Biol Phys. (2023) 117:571–80. doi: 10.1016/j.ijrobp.2023.04.030 37150264 PMC11070071

[13] Lehrer EJ Kowalchuk RO Gurewitz J Bernstein K Kondziolka D Niranjan A . Concurrent administration of immune checkpoint inhibitors and single fraction stereotactic radiosurgery in patients with non-small cell lung cancer, melanoma, and renal cell carcinoma brain metastases. Int J Radiat Oncol Biol Phys. (2023) 116:858–68. doi: 10.1016/j.ijrobp.2023.01.017 36690161

[14] Lehrer EJ Khosla AA Ozair A Gurewitz J Bernstein K Kondziolka D . Immune checkpoint inhibition and single fraction stereotactic radiosurgery in brain metastases from non-small cell lung cancer: an international multicenter study of 395 patients. J Neuro-Oncol. (2023) 165:63–77. doi: 10.1007/s11060-023-04413-4 37889444

[15] Burke AM Carrasquilla M Jean WC Collins BT Anaizi AN Atkins MB . Volume of disease as a predictor for clinical outcomes in patients with melanoma brain metastases treated with stereotactic radiosurgery and immune checkpoint therapy. Front Oncol. (2022) 11:794615. doi: 10.3389/fonc.2021.794615 35096594 PMC8789649

[16] Yang Y Deng L Yang Y Zhang T Wu Y Wang L . Efficacy and safety of combined brain radiotherapy and immunotherapy in non-small-cell lung cancer with brain metastases: a systematic review and meta-analysis. Clin Lung Cancer. (2022) 23:95–107. doi: 10.1016/j.cllc.2021.06.009 34284948

[17] Ciérvide R Martí J López M Hernando O Prado A Alonso L . Single and multitarget stereotactic radiosurgery (SRS) with single isocenter in the treatment of multiple brain metastases (BM): institutional experience. Clin Transl Oncol. (2025) 27:3183–97. doi: 10.1007/s12094-024-03844-3 39814975

[18] Tonse R Tom MC Mehta MP Ahluwalia MS Kotecha R . Integration of systemic therapy and stereotactic radiosurgery for brain metastases. Cancers (Basel). (2021) 13:3682. doi: 10.3390/cancers13153682 34359583 PMC8345095

[19] Deng H Xiong B Gao Y Wu Y Wang W . Stereotactic radiosurgery combined with immune checkpoint inhibitors for brain metastasis: a systematic review and meta-analysis. Asian J Surg. (2023) 46:1917–23. doi: 10.1016/j.asjsur.2022.09.080 36207214

[20] Schulz TU Zierold S Sachse MM Pesch G Tomsitz D Schilbach K . Corrigendum to "Persistent immune-related adverse events after cessation of checkpoint inhibitor therapy: Prevalence and impact on patients' health-related quality of life" [Eur J Cancer 176 (2022) 88-99. Eur J Cancer. (2025) 217:115236. doi: 10.1016/j.ejca.2025.115236 39837704

[21] Bryant A Hiu S Kunonga PT Gajjar K Craig D Vale L . Impact of residual disease as a prognostic factor for survival in women with advanced epithelial ovarian cancer after primary surgery. Cochrane Database Syst Rev. (2022) 9:CD015048. doi: 10.1002/14651858.CD015048.pub2 36161421 PMC9512080

[22] Williams GJ Hong AM Thompson JF . Treatment of melanoma brain metastases with radiation and immunotherapy or targeted therapy: a systematic review with meta-analysis. Crit Rev Oncol Hematol. (2024) 202:104462. doi: 10.1016/j.critrevonc.2024.104462 39097248

[23] Carron R Gaudy-Marqueste C Amatore F Padovani L Malissen N Balossier A . Stereotactic radiosurgery combined with anti-PD1 for the management of melanoma brain metastases: a retrospective study of safety and efficacy. Eur J Cancer. (2020) 135:52–61. doi: 10.1016/j.ejca.2020.04.028 32535348

[24] Brown PD Gondi V Pugh S Tome WA Wefel JS Armstrong TS . Hippocampal Avoidance During Whole-Brain Radiotherapy Plus Memantine for Patients With Brain Metastases: Phase III Trial NRG Oncology CC001. Journal of Clinical Oncology. (2020) 38(10):1019–1029. doi: 10.1200/JCO.19.02767 32058845 PMC7106984

