# Supplementary material for: Stereotactic versus hippocampal avoidance whole-brain radiotherapy combined with immune checkpoint inhibitors for multiple brain metastases in non-small cell lung cancer: a multi-center retrospective study
Source: Front Oncol. 2026 Jul 20;16:1757781. doi: 10.3389/fonc.2026.1757781 (PMC13429507; doi:10.3389/fonc.2026.1757781)
Supplement: Supplementary file 1 [file Table1.docx]

**Table S1** In the matched datasets, the sites of first intracranial progression between the cohorts

| Variable | NR n(%) | LR n(%) | DR n(%) | LR and DR n(%) | P |
| --- | --- | --- | --- | --- | --- |
| SRT+ICIs | 13(21.0) | 4(6.4) | 39(62.9) | 6(9.7) | 0.318 |
| HA-WBRT+ICIs | 11(17.7) | 10(16.1) | 33(53.2) | 8(12.9) |  |

SRT stereotactic radiotherapy, ICI Immune Checkpoint Inhibitor, HA-WBRT hippocampal avoidance whole-brain radiation therapy,NR No recurrences, LR Local recurrences, DR distant recurrences
